# A multicentre phase 1b/2 study of tivozanib in patients with advanced inoperable hepatocellular carcinoma

**DOI:** 10.1038/s41416-020-0737-6

**Published:** 2020-02-10

**Authors:** Christos Fountzilas, Medhavi Gupta, Sunyoung Lee, Smitha Krishnamurthi, Bassam Estfan, Katy Wang, Kristopher Attwood, John Wilton, Robert Bies, Wiam Bshara, Renuka Iyer

**Affiliations:** 1 Roswell Park Comprehensive Cancer Center, Buffalo, NY USA; 2 MD Anderson Cancer Center, Houston, TX USA; 3 Cleveland Clinic, Cleveland, OH USA; 4 University at Buffalo, Buffalo, NY USA

**Keywords:** Hepatocellular carcinoma, Predictive markers

## Abstract

**Background:**

Hepatocellular carcinoma (HCC) is a major cause of cancer-related death. It is a highly vascular tumour with multiple angiogenic factors, most importantly vascular endothelial growth factor (VEGF), involved in HCC progression. Tivozanib is an oral inhibitor of VEGFR-1/2/3 with promising activity against HCC in vivo.

**Methods:**

We conducted a phase 1b/2 study of tivozanib in patients with advanced HCC. The safety, dosing, pharmacokinetics, pharmacodynamics, and preliminary antineoplastic efficacy of tivozanib were evaluated.

**Results:**

Twenty-seven patients received at least one dose of tivozanib. Using a 3+3 design, the recommended phase 2 dose (RP2D) of tivozanib was determined to be 1 mg per os once daily, 21 days on–7 days off. The median progression-free and overall survival were 24 weeks and 9 months, respectively, for patients treated at RP2D. The overall response rate was 21%. Treatment was well tolerated. A significant decrease in soluble plasma VEGFR-2 was noted, assuring adequate target engagement.

**Conclusions:**

Although this study did not proceed to stage 2, there was an early efficacy signal with a very favourable toxicity profile. A phase 1/2 trial of tivozanib in combination with durvalumab is currently underway.

**Trial registration:**

ClinicalTrials.gov NCT01835223, registered on 15 April 2013.

## Background

Hepatocellular carcinoma (HCC) is a major cause of cancer-related death worldwide.^1^ The incidence of HCC is rapidly rising in the United States with a 43% increase in HCC-related mortality over the past two decades.^2^ Patients with advanced (limited to the liver but not amenable to transarterial therapies or metastatic) disease at the time of presentation are not candidates for curative therapy, such as surgical resection, liver transplantation, or tumour ablation.^3^ The lethality of HCC stems in part from its resistance to existing anti-cancer agents, a lack of biomarkers that can detect early disease, and underlying liver disease that limits the use of antineoplastic agents.

HCC is a highly vascular tumour with multiple angiogenic factors, such as vascular endothelial growth factor (VEGF), platelet-derived growth factor (PDGF) and stem cell receptor factor (c-kit), all of which are involved in HCC progression, with VEGF predominating.^4^ Elevated VEGF levels correlate with advanced stage and poor prognosis in HCC.^5^ Sorafenib, a small-molecule inhibitor of VEGFR-1/2/3, BRAF and PDGFR was the first Food and Drug Administration (FDA)-approved oral angiogenesis inhibitor for previously untreated advanced HCC patients with established overall survival (OS) benefit over placebo in large, randomised phase 3 trials.^6,7^ Despite improvement in OS, objective responses are rare (2% or less) and less than half of the patients have meaningful long-term disease control with sorafenib.^7^ For almost a decade, sorafenib remained the only systemic treatment option in advanced HCC. Since the completion of the landmark sorafenib SHARP study, multiple novel multi-receptor or selective receptor kinase inhibitors failed to improve outcomes compared to sorafenib as first-line treatment of advanced HCC.^8–11^ Targeting angiogenesis remains central in the management of advanced HCC, but better agents and biomarkers that define subsets of patients who are likely to derive the most benefit from such therapies are still needed.

Tivozanib is an oral inhibitor of VEGFR-1/2/3, with lesser effects on c-kit and PDGFRβ.^12^ In vitro, it inhibits VEGFR-2, c-kit and PDGFRβ phosphorylation at nanomolar concentrations (half-maximal inhibitory concentration = 0.16, 1.63 and 1.72 nmol/L, respectively).^12^ In vivo, it has dose-dependent activity against HCC^12^ that varies based on vascular phenotype.^13^ Specifically, tivozanib can inhibit tumour growth by almost 60% with daily oral administration at the dose of 0.2 mg/kg for 2 weeks in an immunosuppressed HCC animal model (SK-HEP-1 cells in athymic rats), while at the 1 mg/kg dose tumour inhibition approaches 90%.^12^ Tivozanib was well tolerated at therapeutic doses. A phase 1 study of tivozanib in patients with advanced, refractory solid malignancies established 1.5 mg once daily dosing on a 4 weeks on–2 weeks off schedule as the maximum-tolerated dose (MTD) and recommended phase 2 dose (RP2D) with a manageable toxicity profile.^14^ In a phase 3 trial (TIVO-1), progression-free survival (PFS) and overall response rate (ORR) were superior with tivozanib vs. sorafenib in patients with metastatic renal cell carcinoma (PFS 11.9 vs. 9.1 months, *P* = 0.042; ORR 33.1% vs. 23.3%, *P* = 0.014).^15^

Tivozanib has several advantages over sorafenib. First, it is a potent, highly selective inhibitor of VEGFR-1/2/3 designed to optimise the blockade potential while minimising off-target effects^12^ and has a significantly longer half-life compared to sorafenib (3.7–4.7 days vs. 25–48 h) allowing once daily dosing.^14^ Second, in contrast to sorafenib, tivozanib has no interaction with CYP3A4 inhibitors. Third, it has no associated liver toxicities or tissue accumulation. The improved safety profile of tivozanib over sorafenib was confirmed in the TIVO-1 trial, with hypertension being the predominant toxicity (44% with tivozanib vs. 34% with sorafenib) and lower incidence of off-target skin and gastrointestinal (GI) toxicities (palmar–plantar erythrodysesthesia 14% vs. 54%; diarrhoea 23% vs. 33%) compared to sorafenib.^15^

Based on the preclinical efficacy of tivozanib in in vivo HCC models, the tolerability in early clinical trials, success of targeting angiogenesis in HCC and significantly improved antineoplastic activity in other highly vascular tumours compared to sorafenib, we hypothesised that tivozanib may have meaningful clinical benefit in patients with advanced HCC. In order to test our hypothesis, we conducted an open-label phase 1b/2 trial to assess the safety and efficacy of tivozanib in patients with advanced HCC. Herein, we report the final results of this study.

## Methods

### Study design

This was a phase 1b/2, open-label, single-arm multicentre clinical trial. Subjects were accrued at Roswell Park Comprehensive Cancer Centre (RPCI) and Case Comprehensive Cancer Centre. The respective institutional review board (IRB) approved the protocol at each site. The study was conducted according to the principles of the Declaration of Helsinki, the International Conference on Harmonisation and the Guidelines for Good Clinical Practice. All patients provided written informed consent. The trial was registered with ClinicalTrials.gov (NCT01835223).

Based on available data at the time of study inception, the RP2D of tivozanib was 1.5 mg orally daily. Since tivozanib had not been tested in HCC patients before and considering the underlying cirrhosis present in most of these patients, we decided to test safety first in the phase 1b portion of the study. We followed a 3+3 design, starting at 1 mg (dose level 1, DL1) with planned escalation to 1.5 mg (DL2) and allowing de-escalation to 0.5 mg (DL1). Dose-limiting toxicity (DLT) was defined as any grade 3 or higher toxicity that was possibly, probably, or definitely related to tivozanib. The DLT evaluation period was cycle 1 (28 days). The MTD was defined as the dose level where DLTs were observed in at most one of six patients completing at least one treatment cycle at that dose level. The RP2D was defined as the MTD. The phase 2 portion followed a two-stage design.

### Study population

Eligible patients had metastatic or advanced HCC with either disease progression or ineligibility for surgical resection or locoregional therapies, which was confirmed either by pathological analysis or non-invasively in accordance with American Association for the Study of Liver Diseases criteria,^16^ measurable disease using the RECIST 1.1,^17^ Eastern Cooperative Oncology Group (ECOG) performance status 0–2, Child–Pugh liver function class A, and preserved liver [aspartate aminotransferase ≤5 times institutional upper limits of normal (ULN), total bilirubin ≤3 mg/dL, serum albumin >2.8 g/dL], bone marrow (haemoglobin ≥8.5 g/dL, absolute neutrophil count >1200/mm^3^, platelets ≥60,000/mm^3^), and renal function (creatinine ≤1.5 times institutional ULN). INR had to be ≤2.0. Patients with hepatitis B or C infection were eligible for participation. Key exclusion criteria included prior systemic therapy for HCC, liver transplantation, immunosuppressive therapy, symptomatic or uncontrolled brain metastases or epidural disease and HIV disease.

### Study procedures

Tivozanib was given orally once daily, with or without food for 21 days followed by 7 days off in a 28-day cycle. Tivozanib was administered 1 h before food in a 4 weeks on–2 weeks off schedule in the prior phase 1 study.^14^ The treatment schedule was modified as there was concern on tolerability of longer dosing in patients with underlying cirrhosis. Further, subsequent studies in healthy volunteers did not show a difference in total drug exposure with food consumption^18^ and the administration of oral agents with food can improve the GI toxicity profile. Study treatment was continued until disease progression or unacceptable toxicity. In the case of grade 3 or higher treatment-related adverse events (AEs), tivozanib dosing was interrupted until resolution of toxicity to grade 1 or less and then resumed with dose decreased by 0.5 mg based on pre-specified criteria (see Supplementary Table 1). If tivozanib could not be restarted within 2 weeks, the patient was taken off study treatment. Patients on the phase 1b part with DLT could remain on study and continue treatment at the immediate lower DL upon resolution of toxicity.

Safety assessments were done at baseline, on cycle 1 days 1 and 15, and on day 1 of each cycle thereafter. Tumour assessments were performed by computed tomography or magnetic resonance imaging at baseline and every 8 weeks thereafter. Confirmatory imaging was performed within 6 weeks in patients with complete response or partial response. AEs were assessed using the National Cancer Institute Common Terminology Criteria for Adverse Events (CTCAE) v4.0. Blood sample collection for pharmacokinetic (PK) and pharmacodynamic (PD) analysis was performed on cycle 1 days 1 and 15 pre-dose as well as 2 and 4 h post dose.

### Study objectives

The primary objectives were to determine the MTD/RP2D (phase 1b) and antineoplastic efficacy (phase 2) of tivozanib in patients with HCC. Secondary objectives included determination of safety and the PK/PD of tivozanib in patients with advanced HCC. The primary endpoint was PFS at 24 weeks. PFS and not ORR were selected because at the time of study inception PFS or time to progression were acceptable primary efficacy endpoints in early phase HCC studies in view of very low ORR with anti-VEGFR agents. Patients who remained alive and without evidence of disease progression per RECIST 1.1 for at least 24 weeks after enrolment were considered as PFS responders. Secondary endpoints were AEs by CTCAE v4.0, steady state PK, ORR, OS, change in HBV or HCV viral load, serum α-fetoprotein (AFP), and changes in soluble VEGFR-2 (sVEGFR-2).

### PK and PD analyses

EDTA plasma samples were analysed for tivozanib using a modification of a high-pressure liquid chromatographic (HPLC) assay with tandem mass spectrometric detection (LC-MS/MS)^14^ that was validated according to FDA guidance. Plasma samples (100 μL) were prepared using a protein precipitation procedure and quantitated for tivozanib over a calibration range of 0.500–150 ng/mL. LC-MS/MS analysis of the extracted samples was performed using an Agilent Technologies (Santa Clara, California) HPLC system and the API 3000 mass spectrometer (Sciex, Framingham, MA) with electrospray ionisation. Chromatographic separation was achieved using a biphasic gradient over a Cortecs C18 column (2.1 × 50 mm^2^, Waters Corporation, Milford, MA) preceded by a Cortecs C18+VanGuard Cartridge (2.1 × 5 mm^2^, Waters Corporation). Analytes were detected using multiple reaction monitoring in positive ion mode. Parent/fragment ion pair transitions were 455.20 → 357.10*m*/*z* for tivozanib and 417.10 → 358.00*m*/*z* for KRN-633 (internal standard). Retention times were ~2.76 min for tivozanib and ~2.39 min for KRN-633 with a total run time of 8.5 min/sample. Assay performance was based on tivozanib plasma quality controls (QCs) (*n* = 3 runs) and had an overall accuracy (% analytical recovery) of 108% (104–111%) and overall precision (%RSD) of 3.86% (3.04–4.53%).

A nonlinear mixed-effects model was developed using first-order conditional estimation with interaction (FOCE-I) with the Laplacian option selected. This estimator was chosen as it is more suitable to modelling sparse data sets. One- and two-compartment structural models were tested with first-order absorption, both with and without a separate bioavailability term. Estimates for inter-subject variability were tested on all PK parameters using exponential relationships and were included where they could be confidently estimated. Inter-occasion variability between cycle 1 day 1 and cycle 1 day 15 was also tested. The residual unexplained variability in drug concentrations was tested with additive, proportional, and combined additive and proportional error models. Model selection was performed on the basis of the negative log-likelihood objective function (OFV) and standard goodness-of-fit and diagnostic plots. The final model was composed of one disposition compartment with first-order absorption and elimination. The area under the curve (AUC) of the concentration–time profile was calculated for each individual from 0 to 4 h on days 1 and 15 using the linear trapezoidal method, a form of non-compartmental analysis (NCA). To facilitate comparison with previous studies, AUCs from 0 to 4 and 0 to 24 h were calculated from each individual’s model-predicted PK profile. Similarly, the maximum concentration (*C*_max_) and time of maximum concentration in a given dosing interval (*t*_max_) were estimated both from the original data and the model prediction. The analysis was performed with NONMEM 7.4.1 (ICON plc, Dublin, Ireland), integrated with PSN 4.6.0 (psn.sourceforge.net) and using the Pirana 2.9.2 interface (pirana-software.com).

For sVEGFR-2, plasma EDTA samples (220 μL) were analysed in duplicate using a commercially available enzyme-linked immunosorbent assay (Quantikine® ELISA Human VEGFR-2/KDR immunoassay, R&D Systems® Inc., Minneapolis, MN) that was validated according to the FDA guidance. A Tomtec Quadra 4 (Tomtec Inc., Hamden, CT) liquid handling robot was used to transfer all solutions to the 96-well plate to eliminate time-dependent variability associated with manual pipetting. After the addition of the Stop Solution in the final step, the absorbance of each well was measured at 450 and 540 nm on a Synergy HT plate reader (BioTek Instruments, Winooski, VT). The readings at 540 nm were subtracted from the absorbance readings at 450 nm to correct for any optical imperfections in the 96-well plate. Sample concentrations were back-calculated against calibration curves (80.0–5000 pg/mL) that were generated based on a 4-parameter fit of absorbance vs. nominal concentrations (pg/mL) using the Gen 5 software (BioTek Instruments). Overall assay performance of the sVEGFR-2 assay (*n* = 5 runs) is based on the QCs, which had an overall accuracy of 107% (107–108%) and an overall mean precision of 5.67% (4.66–6.90%).

### Statistical analysis

Using the 3+3 design, the MTD was the DL with probability of DLT 33% or higher. With three dose levels, between 6 and 18 patients were required to complete the phase 1b portion. In terms of antineoplastic efficacy, the true progression rate of less than *p*0 = 0.50 was considered unacceptable and evidence of such will deem the treatment not worthy of further study. The null and alternative hypotheses tested were H0: *p* = *p*0 vs. H1: *p* > *p*0. With this two-stage design, a potential total of 37 patients was required to achieve 80% power to detect a difference of at least 20% with a type I error of 5%. Using a two-stage design, a total of 19 patients were planned to be enrolled initially, including the phase 1b patients treated at the MTD/RP2D. If nine or fewer PFS responses were observed, the therapy would be deemed ineffective and the study would end. Otherwise, an additional 18 patients would be enrolled. If 23 or fewer PFS responses are observed in the total efficacy population, therapy would be deemed ineffective; otherwise, it will be concluded that the therapy is promising. The differences on outcomes between study groups were compared by non-parametric Kruskal–Wallis test and Fisher’s/*χ*^2^ test. OS and PFS were estimated by the Kaplan–Meier method and compared by log-rank test. All tests were two sided and performed at a nominal significance level of 0.05. SAS version 9.4 (SAS Institute, Cary, NC) is used for statistical analyses.

## Results

### Patients

Between July 2013 and November 2016, 33 patients were consented for participation in three sites. The 27 patients who were deemed eligible and received at least one dose of tivozanib are included in the safety analysis, the 19 patients who were treated at the RP2D and were evaluable for the primary efficacy endpoint are included in the efficacy analysis. Six patients were not evaluable for efficacy as they either rapidly progressed clinically (*n* = 3) or withdrew consent (*n* = 2), one patient was on treatment for 13 days only and was taken off study secondary to kidney dysfunction and precluding end of treatment imaging. The cut-off date for the efficacy analysis was 31 March 2019. Patient demographics, baseline disease characteristics and previous treatments are summarised in Table 1. The median follow-up for all patients in the efficacy population was 37.3 months (range: 5.4–209.9). Six patients had prior tumour ablation and one patient had prior transarterial chemoembolization.

**Table 1:** Baseline demographics, efficacy population (*N* = 19).

Age in years, median (range)	67.5 (23.3–81.8)
Gender, *n* (%)	
Male	19 (100)
Female	0 (0)
Extrahepatic disease, *n* (%)	
No	4 (21)
Yes	15 (79)
ECOG PS, *n* (%)	
0	12 (63)
1	7 (37)
Baseline serum albumin in g/dl, median (range)	4.0 (3.3–4.7)
Baseline serum bilirubin in mg/dl, median (range)	0.9 (0.3–1.9)
Baseline serum AFP in ng/ml, median (range)	394.4 (3.3–200,000.0)

*AFP* α-fetoprotein, *ECOG* Eastern Cooperative Oncology Group, *PS* performance status.

### Phase 1b

A total of eight DLT-evaluable patients were enrolled in the phase 1b portion. The first three patients were enrolled at DL1 (1 mg) with no DLT. The next two patients were enrolled at the DL2 (1.5 mg) and both experienced DLT (one with grade 3 stomatitis and one with grade 3 hypertension). These patients continued at DL1 after resolution of toxicity per protocol without further DLT. Three more patients were enrolled at DL1 with one experiencing DLT (grade 3 palmar–plantar erythrodysesthesia). Therefore, 1 mg once daily was determined to be the MTD/RP2D.

### Phase 2

**Fig. 1:**
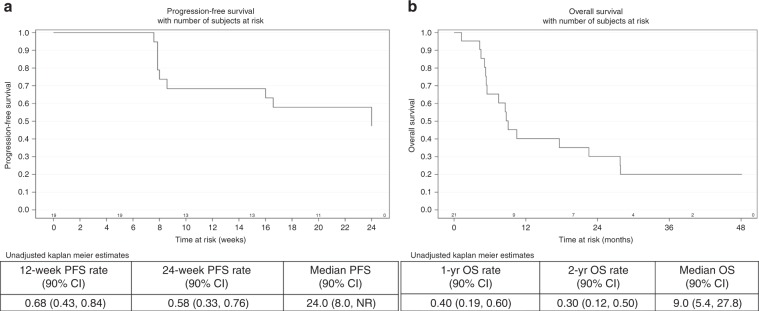
Progression-free and overall survival in efficacy population. Progression-free (**a**) and overall survival (**b**) in efficacy population.

Thirteen more evaluable patients were enrolled at DL1 and the efficacy analysis was conducted on a total of 19 patients. All patients are off study at the time of this report, one patient came off study due to a coronary event after over 3 years and has no measurable disease 18 months after stopping tivozanib with no additional anti-tumour therapy. There were nine PFS responders at 24 weeks (47.3%; 90% confidence interval (CI) 27–68). The median PFS was 24 weeks with a 24-week PFS probability of 58% (90% CI: 33–76, Fig. 1a). The study stopped accrual for futility per the pre-specified statistical plan. Notably, four patients had no evidence of disease progression for more than 2 years. After a median follow-up of 37.3 months, the median OS was 9.0 months (Fig. 1b). The 1- and 2-year OS rate were 40% (90% CI: 19–60) and 30% (90% CI: 12–50%), respectively. Three patients are alive at the time of this report.

**Fig. 2:**
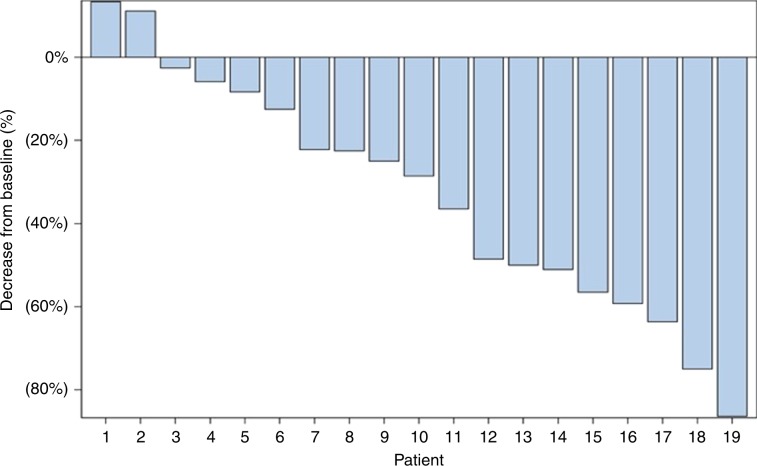
RECIST response, waterfall plot.

Four patients attained a PR (21%) and eight (42%) had stable disease (SD), for an overall disease control rate of 63% (Fig. 2). Most patients had evidence of tumour shrinkage on imaging. Eighteen patients had baseline and on-treatment serum AFP measurements. Four patients had AFP decrease >50%. Patients with AFP decrease >50% had longer PFS and OS; however, these measures did not reach statistical significance (Supplementary Fig. 1). Seven out of 12 patients with ECOG performance status 0 were progression free at 24 weeks as compared to two out of seven patients with performance status of 1 (*P* = 0.34).

### Safety

Overall, tivozanib was well tolerated. Of the patients evaluable for safety (*n* = 27), six had dose reductions for toxicity, 12 patients had temporary tivozanib discontinuation, and four patients were permanently discontinued from study treatment for toxicity. Treatment-related AEs are presented in Table 2. Fatigue (63%, grade 3–4: 26%), diarrhoea (41%, grade 3–4: 0%), decreased appetite (37%, grade 3 and 4: 3.7%), nausea (26%, grade 3 and 4: 0%), dysphonia (26%, grade 3 and 4: 0%), vomiting (22%, grade 3 and 4: 0%), stomatitis (18.5%, grade 3 and 4: 0%), increased bilirubin (18.5%, grade 3 and 4: 7.4%) and hypertension (18.5%, grade 3 and 4: 11%). The incidence of palmar–plantar erythrodysesthesia was 11% (grade 3 and 4: 3.7%). The incidence of grade 3 pulmonary embolism was 11%. Serious AEs (regardless of causality) are presented in Supplement Table 2. No deaths due to toxicity were seen. Viral loads were followed for two HBV-positive and three HCV-positive patients and remained stable (data not shown).

**Table 2:** Adverse events possibly, probably or definitively related to tivozanib (*n*, %).

	Grade 1		Grade 2		Grade 3		Grade 4		Grade 5		Total	
Fatigue	6	22	4	15	7	26	0	0	0	0	17	63
Diarrhoea	6	22	5	19	0	0	0	0	0	0	11	41
Decreased appetite	3	11	6	22	1	4	0	0	0	0	10	37
Nausea	4	15	3	11	0	0	0	0	0	0	7	26
Dysphonia	6	22	1	4	0	0	0	0	0	0	7	26
Vomiting	4	15	2	7	0	0	0	0	0	0	6	22
Stomatitis	4	15	1	4	0	0	0	0	0	0	5	19
Elevated bilirubin	0	0	3	11	2	7	0	0	0	0	5	18
Hypertension	1	4	1	4	2	7	1	4	0	0	5	19
Thrombocytopenia	4	15	0	0	0	0	0	0	0	0	4	15
Increased ALP	0	0	0	0	3	11	0	0	0	0	3	11
Lymphocytopenia	1	4	1	4	1	4	0	0	0	0	3	12
Epistaxis	3	11	0	0	0	0	0	0	0	0	3	11
Pulmonary embolism	0	0	0	0	3	11	0	0	0	0	3	11
PPEDS	2	7	0	0	1	4	0	0	0	0	3	11
Increased ALT	1	4	0	0	1	4	0	0	0	0	2	8
Decreased weight	0	0	1	4	1	4	0	0	0	0	2	8
Dizziness	2	7	0	0	0	0	0	0	0	0	2	7
Headache	2	7	0	0	0	0	0	0	0	0	2	8
Dry skin	1	4	1	4	0	0	0	0	0	0	2	7
Pruritus	1	4	1	4	0	0	0	0	0	0	2	8

*ALP* alkaline phosphatase, *ALT* alanine aminotransferase, *PPEDS* palmar–plantar erythrodysesthesia syndrome.

### Pharmacokinetics

All 27 patients who received at least one dose of tivozanib are included in the PK analysis. The lower limit of quantitation for tivozanib was 0.500 ng/mL in plasma. Only one post-dose below the lower limit of quantitation (BLQ) observation was present in the dataset. BLQ observations were treated as censored data using the M3 method.^19^ Parameter estimates for the population can be found in Supplementary Table 3. Bioavailability could not be reliably characterised, so parameters are reported as relative to bioavailability. It should be noted that although a one-compartment disposition model fit the data best, the concentration data may have been too sparse to detect a two-compartment disposition. Inter-individual variability was estimated for the absorption rate constant (ka), systemic clearance (CL), and volume of distribution (*V*). Between-occasion variability in the parameters could not be reliably estimated. A proportional error model best characterised the residual unexplained variability. Individual PK parameter estimates are listed in Supplement Table 4. Population-level NCA and model predictions are presented in Table 3, while the individual exposures are shown in Supplementary Table 5. Mean AUC_0–4_ on days 1 and 15 were 14.28 and 190.06 h*ng/mL in the prediction model, respectively, similar to the NCA model. The predicted AUC_0–24_ on days 1 and 15 were 175.98 and 1104.89 h*ng/mL, respectively. AUCs on day 15 were higher compared to day 1 because of expected accumulation. There was no significant difference between the observed and predicted *C*_max_ on days 1 and 15. However, observed *t*_max_ was lower compared to predicted (median day 1: 4.07 vs. 13.33 h; median day 15: 2.05 vs. 7 h).

**Table 3:** Population secondary pharmacokinetic parameters.

Day	Dose (mg)	AUC_0–4_ (h*ng/mL)		AUC_0–24_ (h*ng/mL)		*C*_max_ (ng/mL)		*t*_max_ (h)	
		Mean	Standard deviation	Mean	Standard deviation	Mean	Standard deviation	Median	Range
NCA and observation									
1	1	14.22	7.87	NE	NE	6.54	3.82	4.067	2.05–7.67
15	1	184.33	98.14	NE	NE	58.67	36.84	2.05	2.00–4.12
Model prediction									
1	1	14.28	7.66	175.98	74.29	8.75	3.27	13.33	9.63–23.97
15	1	190.06	90.79	1104.89	589.09	50.67	23.35	7.00	5.97–17.93

*NCA* non-compartmental analysis*, AUC*_*0–4*_ and *AUC*_*0–24*_ area under the concentration–time curve from 0 to 4 and 0 to 24 h, respectively, *NE* not estimated.

### Pharmacodynamics

**Fig. 3:**
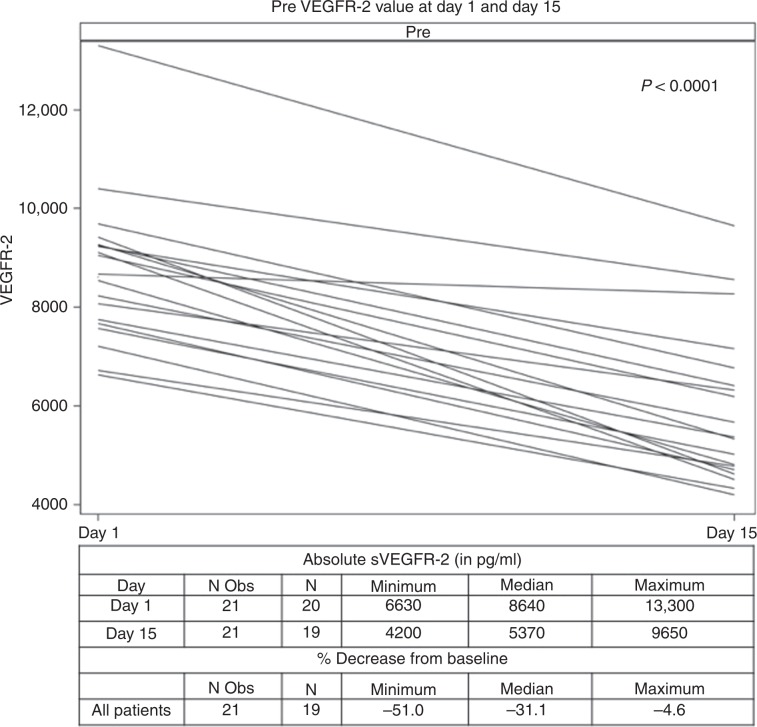
Pre-dose sVEGFR-2, cycle 1 days 1 and 15.

The level of sVEGFR-2 decreased between days 1 and 15 (median pre-dose levels 8.64 µg/L on day 1 and 5.37 µg/L on day 15, *P* < 0.0001; Fig. 3). The decrease in sVEGFR-2 by PFS and RECIST response is shown in Supplement Fig. 2. The median percent decrease from baseline was 31.1% in all patients, 32.4% vs. 30.1% (*P* = 0.3359) in PFS responders versus not, and 27.4% vs. 40.5% vs. 30.9% in progressive disease (PD), SD and PR by RECIST 1.1, respectively (*P* = 0.0346).

## Discussion

This is to our knowledge the first reported study with tivozanib in patients with advanced HCC. The MTD and RP2D was established at 1 mg once daily for 21 days, followed by 7 days of rest. The toxicity profile was manageable. Compared to patients with all solid tumours treated at the 1 mg dose level, there was higher incidence of fatigue (any grade: 63% vs. 55%; grade 3–4: 26% vs. 6%).^14^ Interestingly, we observed a lower incidence of palmar–plantar erythrodysesthesia (any grade: 11% vs. 22%), hypertension (any grade: 19% vs. 39%; grade 3–4: 11% vs. 28%) and dry skin (any grade: 7% vs. 28%); GI toxicity was similar. Compared to patients with renal cell carcinoma treated with tivozanib at a higher dose (1.5 mg on a 3 week on–1 week off schedule), fatigue and GI AEs are more frequent in HCC, but hypertension is less common; the incidence of palmar–plantar erythrodysesthesia is similar.^15^ With the limitation of the different modelling method used (although there was concordance between the observed and predicted AUC_0–4_), the tivozanib exposure at the 1 mg dose appears higher in patients with HCC compared to patients in the all solid tumour phase 1 study treated at the same dose after single and multiple dosing [mean AUC_0–24_: 175.98 vs. 131.2 h*ng/mL (single dose) and 1104.89 vs. 856 h*ng/mL (multiple doses)].^14^ *C*_max_ appears similar in patients with HCC and all solid tumours using both models, but it appears this is achieved faster in HCC patients [median *t*_max_: 4.07 vs. 6.01 h (single dose) and 2.05 vs. 4.01 h (multiple doses)]. These data underscore the significance of underlying liver disease in tolerance of antineoplastic agents and the importance of phase 1 studies specifically for patients with HCC or various degrees of liver dysfunction.^20^ It is important to note that the realistic eligibility criteria of this study allowed enrolment that is more consistent to real-world HCC patients and thus the RP2D found is more generalisable.

Although the study did not meet the threshold to activate stage 2, the antineoplastic efficacy was promising with 6-month PFS and 1-year OS at 47.3% and 38%, respectively, and ORR of 21% with some very durable responses. At the time of the study inception, sorafenib was the only approved agent for advanced HCC. The landscape since has changed significantly with many options in the first-^21^ and second-line setting.^22–25^ The antineoplastic efficacy of tivozanib compares favourably to sorafenib and lenvatinib as first-line treatment with an ORR (RECIST 1.1) of 21% vs. 18.8% (lenvatinib) and 6.5% (sorafenib)^21^ and is at the same range as immune checkpoint inhibitors in the second line.^24,25^ The percentage of patients who were progression free at 6 months (47.3%) appears to be higher than with sorafenib (35%) and slightly lower than with lenvatinib (55%). Importantly, tolerance appears to be similar to both sorafenib and lenvatinib with no grade 3 or higher GI AEs and very low incidence of clinically significant palmar–plantar erythrodysesthesia and hypertension. A notable exception is fatigue with incidence that appears higher than sorafenib (all grades: 25%; grade 3–4: 4%) or lenvatinib (any grade 30%; grade 3–4: 4%).^21^ This can be potentially attributable to the individual drug on-target/off-tumour activity and/or different potencies against different cellular kinases as tivozanib has an anti-VEGFR-2 activity in vitro that is 187 times higher compared to sorafenib and 32 times higher compared to lenvatinib; its activity against PDGFRβ is 46 and 197 times higher compared to sorafenib and lenvatinib, respectively.^12,26,27^

We demonstrated a consistent reduction in sVEGFR-2 between C1D1 and C1D15 for all patients with a median of 31.1%. Similarly, in the phase 1 study by Eskens et al.,^14^ sVEGFR-2 was decreased on days 15 and 27 in a dose-dependent manner with a 28% decrease from baseline by day 27 in patients enrolled in the 1 mg daily cohort.^14^ Interestingly, in our study, patients with SD had significantly higher percentage decrease on day 15 (40.5%) compared to patients with PD (27.4%) or PR (30.9%). Selective VEGFR-2 inhibition with ramucirumab does not usually lead to objective responses in HCC^28,29^ and it is likely that the inhibition of other tivozanib targets, rather than VEGFR-2, is more important in terms of tumour shrinkage. In addition, we have previously linked higher sVEGFR-2 exposure with increased progression risk on anti-angiogenic agents in HCC.^30^ Similarly, in the current study, the baseline sVEGFR-2 was lower in PFS responders (7.95 vs. 8.86 µg/L in PFS non-responders) and patients with SD or PD had higher baseline levels than patients with PR (9.05 vs. 8.67 µg/L vs. 7.9 µg/L, respectively). The differences are not statistically significant, most likely the result of small sample size. Moreover, we cannot firmly conclude that baseline sVEGFR-2 can be a potential negative predictive biomarker for response to tivozanib. Finally, enrolment of a biologically good prognosis patient population in our study—as the median baseline sVEGFR-2 levels are within the range reported for healthy volunteers (5–10 µg/L) and lower than the one reported for HCC (18.3 µg/L)—cannot be excluded.^30–32^

In an exploratory post hoc analysis in six patients with adequate quality tissue for correlative studies, higher baseline CD8^+^ cell infiltration by immunohistochemistry was present in PFS responders (data not shown), results that are in line with prior observations.^33^ Immune dysfunction is a hallmark of advanced HCC^34^ despite the presence of tumour-specific immune responses in some patients.^35^ Previously published data from our group reveal that anti-VEGF agents have immunomodulatory properties that are dose and time dependent. Specifically, sorafenib can inhibit T cell proliferation in higher doses, while in low doses it significantly increases T cell infiltration and inhibits tumour growth.^36^ Further, sorafenib leads to an increase in CD4^+^CD127^+^PD-1^−^ effector T cells relative to CD4^+^Foxp3^+^PD-1^+^ regulatory T cells with increased frequency of the former in post-treatment samples correlating with OS.^37^ With small-molecule multikinase inhibitors able to at least partially reverse immune dysfunction, multiple clinical trials are now evaluating anti-VEGF agents in combination with immune checkpoint inhibitors in advanced HCC and the preliminary results of this strategy are promising.^38,39^ A study of tivozanib in combination with the PD-L1 inhibitor durvalumab is currently underway (NCT03970616).

## Supplementary information

Supplement

## Data Availability

Data can be available upon request.
