# Supplementary material for: A multicentre phase 1b/2 study of tivozanib in patients with advanced inoperable hepatocellular carcinoma
Source: Br J Cancer. 2020 Feb 10;122(7):963–70. doi: 10.1038/s41416-020-0737-6 (PMC7109127; doi:10.1038/s41416-020-0737-6)
Supplement: Supplementary file 1 — Supplement [file 41416_2020_737_MOESM1_ESM.docx]

| Supplement Table 1. Tivozanib Dose Modifications. Dose reductions of tivozanib by 0.5 mg/day was required for patients with ≥ Grade 3 treatment-related adverse events. This excluded grade ≥ 3 hypertension, nausea, vomiting, and diarrhea, without adequate supportive care for which tivozanib could resume at the same dose level after adequate supportive care, if in the investigators opinion it was safe to proceed at the same dose level. If hypertension, diarrhea, nausea, and vomiting recurred despite adequate supportive care tivozanib was dose reduced. Up to 2 dose reductions are allowed. Once a patient’s dose of tivozanib is reduced, it could not be re-escalated. | | |
| --- | --- | --- |
| Treatment-Related Adverse Events | Action Taken | Subsequent Dosing Modification |
| Grade 1 | No dose interruption, or reduction  required; adverse event management is at the discretion of the Investigator | None required; Dosing may continue at the same dose |
| Grade 2 | No dose interruption, or reduction  required; adverse event management is at the discretion of the Investigator | None required; Dosing may continue at the same dose |
| Grade 3 | Interrupt dosing until toxicity resolves to grade < 1 | Dosing may resume at reduced dose |
| Grade 4 | Interrupt dosing until toxicity resolves to grade < 1 | Dosing may resume at reduced dose |

| Supplement Table 2. Serious Adverse Events regardless of causality (n, %) | | | | | | | | | | |
| --- | --- | --- | --- | --- | --- | --- | --- | --- | --- | --- |
|  | Grade 2 | | Grade 3 | | Grade 4 | | Grade 5 | | Total | |
| Pulmonary embolism | 0 | 0 | 2 | 7 | 0 | 0 | 0 | 0 | 2 | 7 |
| Acute myocardial infarction | 0 | 0 | 1 | 4 | 0 | 0 | 0 | 0 | 1 | 4 |
| Blood bilirubin increased | 0 | 0 | 1 | 4 | 0 | 0 | 0 | 0 | 1 | 4 |
| Cerebrovascular accident | 1 | 4 | 0 | 0 | 0 | 0 | 0 | 0 | 1 | 4 |
| Acute kidney injury | 0 | 0 | 0 | 0 | 0 | 0 | 1 | 4 | 1 | 4 |
| Hypertension | 0 | 0 | 0 | 0 | 1 | 4 | 0 | 0 | 1 | 4 |

| Supplement Table 3. Primary PK Parameters estimates | | | |
| --- | --- | --- | --- |
| Parameter | Units | Estimate of Typical Population Value | Relative Standard Error of Estimate (%CV) |
| Apparent Systemic Clearance (CL/F) | L/h | 0.803 | 14.1 |
| Apparent Volume of Distribution (V/F) | L | 105 | 25.9 |
| Apparent first-order absorption rate constant (ka/F) | 1/h | 0.239 | 59.0 |
| Inter-individual variability (IIV) in CL/F | %CV | 42.4 | 50.5 |
| Inter-individual variability (IIV) in V/F | %CV | 45.8 | 78.1 |
| Inter-individual variability (IIV) in ka/F | %CV | 57.5 | 122.7 |
| Proportional residual unexplained variability | %CV | 0.0923 | 24.2 |
| Standard errors were estimated using a statistical bootstrap of 1000 resampled populations. | | | |

| Supplement Table 4. Individual PK parameters | | | |
| --- | --- | --- | --- |
| Subject ID | Apparent systemic clearance (L/h) | Apparent volume of distribution (L) | Apparent first-order absorption rate constant (1/h) |
| 010101RP | 0.81 | 114 | 0.181 |
| 010202RP | 0.80 | 159 | 0.127 |
| 010304RP | 1.00 | 83 | 0.342 |
| 010203RP | 1.59 | 78 | 0.267 |
| 020105RP | 0.80 | 87 | 0.294 |
| 020206RP | 0.56 | 67 | 0.268 |
| 020107RP | 0.28 | 49 | 0.285 |
| 010410RP | 0.48 | 71 | 0.347 |
| 010511RP | 0.80 | 159 | 0.137 |
| 010612RP | 0.75 | 123 | 0.223 |
| 01P213RP | 0.81 | 150 | 0.164 |
| 01P214RP | 1.04 | 100 | 0.256 |
| 01P215RP | 0.81 | 70 | 0.374 |
| 01P216RP | 1.39 | 90 | 0.264 |
| 01P217RP | 1.37 | 95 | 0.284 |
| 01P218RP | 0.62 | 107 | 0.180 |
| 01P219RP | 0.84 | 124 | 0.177 |
| 01P220RP | 0.99 | 96 | 0.256 |
| 01P221RP | 1.22 | 113 | 0.272 |
| 01P222RP | 1.12 | 154 | 0.160 |
| 01P223RP | 0.99 | 108 | 0.287 |
| 01P224RP | 0.67 | 90 | 0.223 |
| 2291CC01 | 0.81 | 206 | 0.097 |
| 2291UH02 | 0.72 | 198 | 0.086 |
| 2291UH03 | 0.82 | 190 | 0.105 |
| 01P225RP | 1.07 | 90 | 0.347 |
| 01P226RP | 0.95 | 93 | 0.278 |

| Supplement Table 5. Individual Tivozanib Exposure | | | | | | | | | | | | | | |
| --- | --- | --- | --- | --- | --- | --- | --- | --- | --- | --- | --- | --- | --- | --- |
| Subject ID | NCA and Observation | | | | | | Model Prediction | | | | | | | |
|  | Day 1 | | | Day 15 | | | Day 1 | | | | Day 15 | | | |
|  | C_max_ (ng/mL) | t_max_ (h) | AUC_0-4_ (h*ng/mL) | C_max_ (ng/mL) | t_max_ (h) | AUC_0-4_ (h*ng/mL) | C_max_ (ng/mL) | t_max_ (h) | AUC_0-4_ (h*ng/mL) | AUC_0-24_ (h*ng/mL) | C_max_ (ng/mL) | t_max_ (h) | AUC_0-4_ (h*ng/mL) | AUC_0-24_ (h*ng/mL) |
| 010101RP | 5.82 | 4.05 | 8.9 | 57.4 | 2.02 | 185.6 | 7.66 | 18.65 | 10.0 | 150.8 | 48.48 | 8.53 | 468.1 | 1713.2 |
| 010202RP | 2.97 | 4.033 | 4.7 | NA | NA | NA | 5.48 | 23.97 | 5.4 | 98.6 | NA | NA | NA | NA |
| 010304RP | 8.63 | 4.167 | 24.5 | 47.3 | 2.01 | 154.1 | 10.68 | 10.15 | 21.6 | 222.6 | 44.11 | 5.97 | 383.0 | 1460.6 |
| 010203RP | 10.6 | 4.05 | 19.4 | 30.8 | 2.07 | 106.3 | 10.37 | 10.45 | 19.2 | 211.2 | 29.49 | 6.52 | 219.5 | 922.5 |
| 020105RP | 8.57 | 4.067 | 20.2 | NA | NA | NA | 10.28 | 12.17 | 18.8 | 214.4 | NA | NA | NA | NA |
| 020206RP | 13.6 | 4.417 | 21.6 | 89.5 | 2.06 | 333.0 | 13.30 | 13.33 | 22.6 | 271.4 | 73.71 | 6.97 | 640.0 | 2542.1 |
| 020107RP | 16.3 | 4.05 | 35.9 | 201 | 2.01 | 558.5 | 18.93 | 14.05 | 32.8 | 395.1 | 134.40 | 7.17 | 1365.7 | 4735.5 |
| 010410RP | 9.53 | 4.067 | 27.7 | 104 | 2.1 | 360.8 | 12.95 | 11.63 | 25.3 | 274.4 | 82.90 | 6.27 | 673.6 | 2872.0 |
| 010511RP | 1.42 | 2.167 | 1.5 | NA | NA | NA | 5.55 | 23.95 | 5.8 | 101.5 | NA | NA | NA | NA |
| 010612RP | 3.62 | 2.167 | 11.0 | 58.8 | 2.03 | 205.7 | 7.36 | 16.63 | 10.8 | 148.3 | 50.43 | 8.00 | 545.6 | 1785.2 |
| 01P213RP | 2.78 | 4.083 | 7.0 | NA | NA | NA | 5.93 | 21.53 | 7.0 | 113.5 | NA | NA | NA | NA |
| 01P214RP | 6.93 | 4.183 | 16.2 | 43.1 | 2.07 | 154.1 | 8.73 | 13.03 | 14.6 | 179.3 | 41.44 | 6.97 | 353.7 | 1409.7 |
| 01P215RP | 11.8 | 4.067 | 30.3 | 47 | 2.12 | 177.6 | 12.73 | 9.63 | 26.7 | 267.7 | 48.61 | 17.93 | 1374.9 | 1537.1 |
| 01P216RP | 8.23 | 4.017 | 18.0 | 31.1 | 2.12 | 112.9 | 9.34 | 11.43 | 16.6 | 192.5 | 32.60 | 6.68 | 226.2 | 1056.3 |
| 01P217RP | 6.82 | 4.067 | 18.1 | 32.3 | 2.01 | 103.0 | 8.99 | 11.03 | 16.5 | 185.8 | 32.48 | 6.52 | 289.9 | 1066.9 |
| 01P218RP | 5.84 | 4.067 | 10.6 | 70.9 | 2.03 | 261.2 | 8.36 | 19.63 | 10.6 | 163.3 | 60.01 | 8.80 | 505.3 | 2131.8 |
| 01P219RP | 4.7 | 4.2 | 10.7 | 61.2 | 2 | 156.5 | 7.06 | 19.20 | 9.0 | 138.7 | 46.41 | 8.67 | 440.3 | 1643.8 |
| 01P220RP | 7.56 | 4.033 | 16.6 | 49.9 | 2.02 | 150.0 | 9.11 | 13.07 | 15.5 | 188.3 | 43.28 | 7.00 | 375.3 | 1473.6 |
| 01P221RP | 4.71 | 2.067 | 13.8 | 33.3 | 4.12 | 134.8 | 7.74 | 12.33 | 13.5 | 160.5 | 34.79 | 6.83 | 378.7 | 1186.1 |
| 01P222RP | 3.4 | 4.2 | 6.8 | 37.8 | 4.09 | 134.3 | 5.60 | 20.18 | 6.7 | 107.0 | 35.12 | 8.88 | 340.2 | 1244.2 |
| 01P223RP | 5.18 | 2.05 | 15.5 | 49.9 | 2.05 | 148.1 | 8.27 | 12.43 | 14.7 | 172.4 | 42.35 | 6.75 | 426.7 | 1454.0 |
| 01P224RP | 8.48 | 4.133 | 10.3 | 69.4 | 2.02 | 239.7 | 9.87 | 15.78 | 14.8 | 199.9 | 59.89 | 7.73 | 528.4 | 2093.8 |
| 2291CC01 | 1.4 | 5.25 | 3.6 | NA | NA | NA | 4.08 | 22.75 | 3.3 | 63.8 | NA | NA | NA | NA |
| 2291UH02 | 0.616 | 7.667 | 0.6 | 53.6 | 2.02 | 175.8 | 3.99 | 20.33 | 3.1 | 52.8 | 42.23 | 13.92 | 482.7 | 1534.3 |
| 2291UH03 | 2 | 4.083 | 3.5 | 43.4 | 3.91 | 163.5 | 4.50 | 23.92 | 3.9 | 76.6 | 40.34 | 11.98 | 305.5 | 1460.0 |
| 01P225RP | 7.29 | 2.1 | 21.5 | 40.3 | 2.1 | 154.7 | 9.84 | 10.10 | 20.0 | 206.0 | 40.91 | 5.98 | 402.4 | 1363.4 |
| 01P226RP | 7.79 | 4.35 | 18.8 | 38.7 | NA | NA | 9.50 | 12.30 | 16.9 | 194.9 | NA | NA | NA | NA |

**
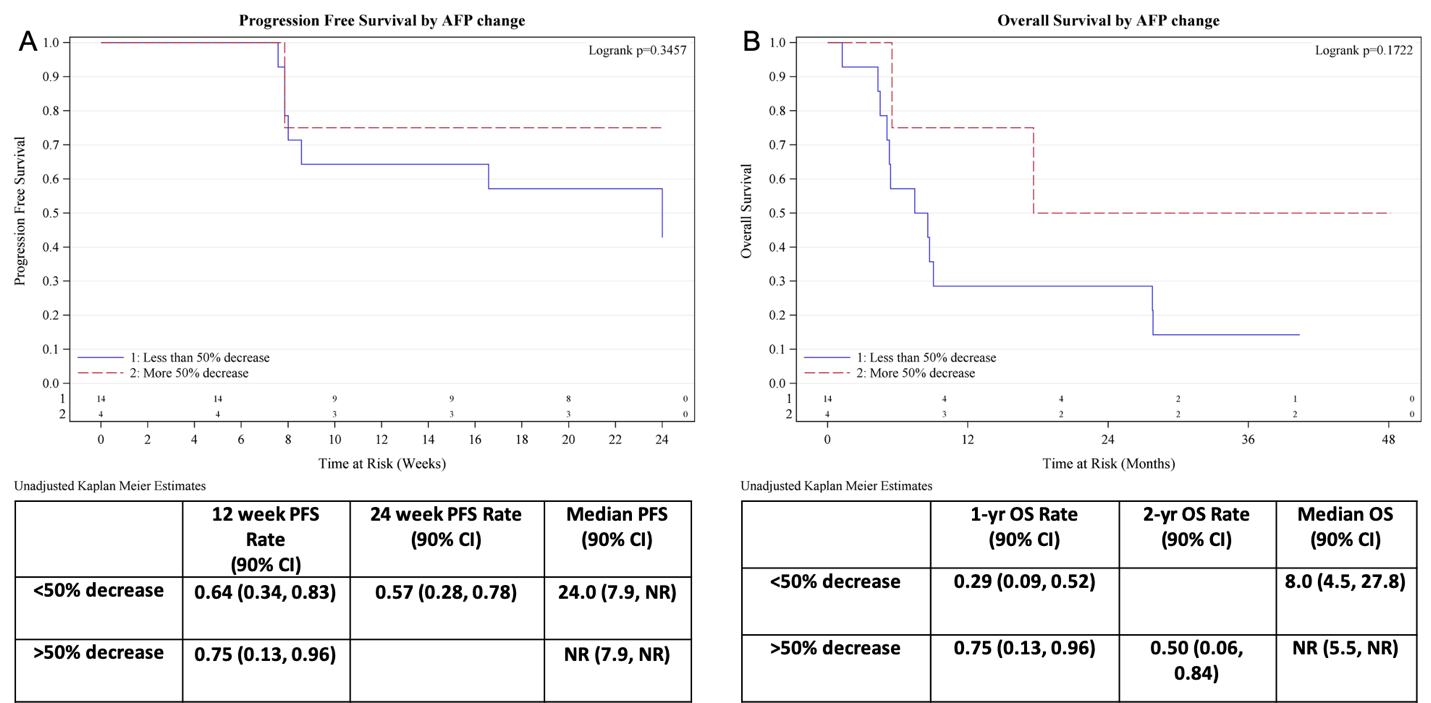
**

**Supplement Figure 1. Progression-free (A) and overall survival by AFP change >50%.**


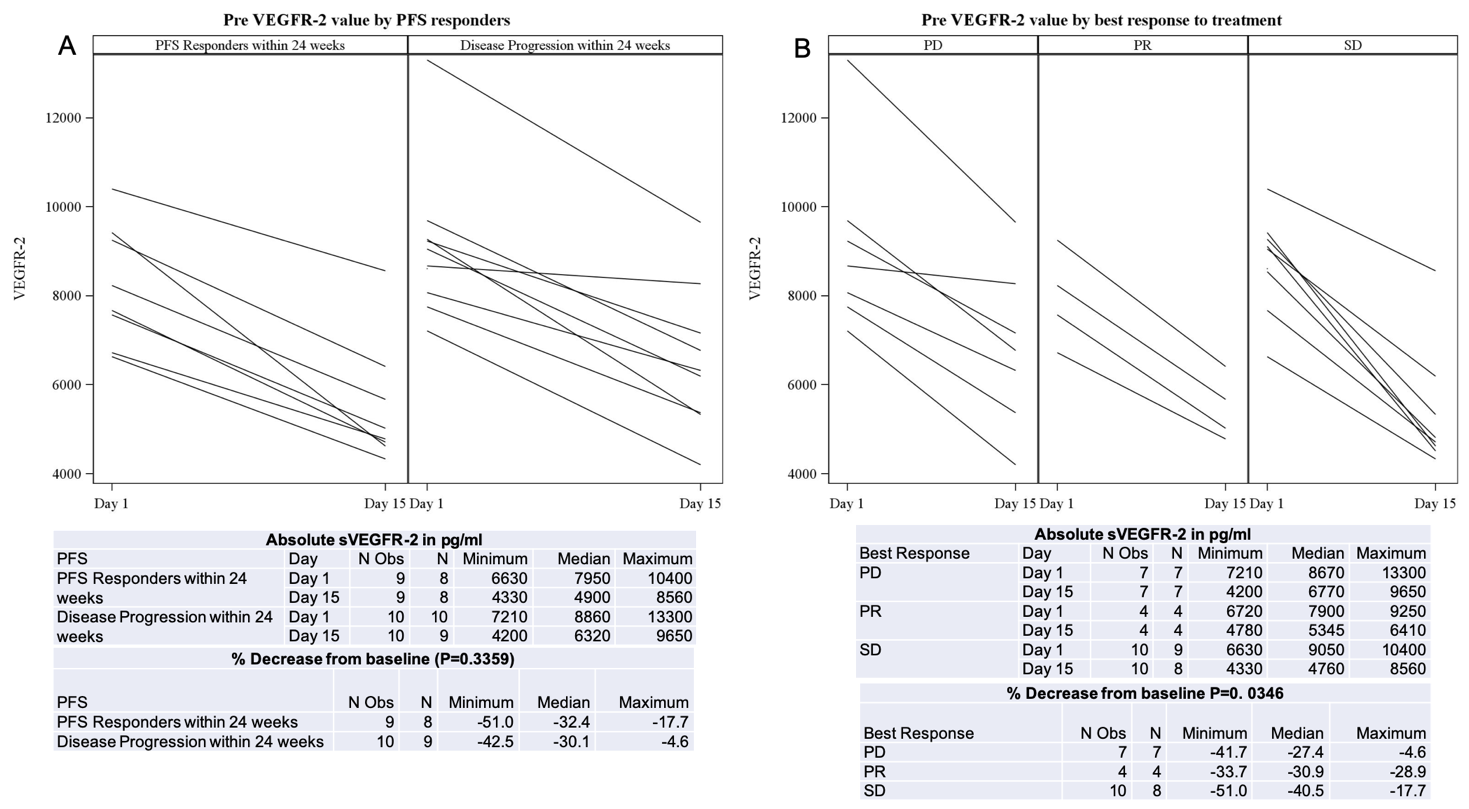


**Supplement Figure 2. sVEGFR on day 1 and day 15 by progression-free survival at 24 weeks (A) and RECIST 1.1 (B) response.**
